# Pituitary stalk management during the microsurgery of craniopharyngiomas

**DOI:** 10.3892/etm.2014.1561

**Published:** 2014-02-19

**Authors:** GELEI XIAO, XIANRUI YUAN, JIAN YUAN, NADEEM AKHTAR KRUMTALLY, YIFENG LI, CHENGYUAN FENG, QING LIU, ZEFENG PENG, XUEJUN LI, XIPING DING

**Affiliations:** Institute of Skull Base Surgery and Neurooncology at Hunan, Xiangya Hospital, Central South University, Changsha, Hunan 410008, P.R. China

**Keywords:** craniopharyngioma, pituitary stalk, microsurgery, recurrence, endocrine function

## Abstract

In the present study, 203 patients that had previously undergone microsurgery for craniopharyngiomas (CPs) between 1992 and 2012 were analyzed retrospectively on a long-term follow-up basis to investigate the differences in the recurrence rate and endocrine function between patients with preserved and resected pituitary stalks. To summarize the possible outcomes of microsurgery, the 203 patients were divided into 2 groups: Group A that had preserved pituitary stalks and Group B that had undergone resections of the pituitary stalk. Tumor origins and the involvement of the pituitary stalk during surgery were observed. From 2010 onwards, an ultra-electron microscope was used postoperatively to detect whether pituitary stalk specimens were infiltrated or invaded with tumor cells. Long-term follow-up observations of the patients included tumor recurrence, postoperative endocrine dysfunction and visual acuity and field. Among the 203 patients, 175 patients received gross-total resection (GTR) (175/203, 86.2%), 28 patients underwent subtotal resection (28/203, 13.8%) and 34 patients had surgery that preserved the pituitary stalk (34/203, 16.7%). There was no significant difference in the recurrence rate between Group A (4/34, 11.8%) and the patients in Group B (10/123, 8.1%) who underwent GTR and also received follow-ups. Of the 157 patients who were followed up, 91 individuals underwent endocrine evaluation and the outcome was divided into normal, satisfactory and poor grades. The results for Group A were 5, 18 and 0, respectively, while the results for Group B were 1, 60 and 7, respectively, which showed a statistically significant difference between the groups. Pituitary stalk specimens of 15 patients were studied postoperatively using an ultra-electron microscope and all samples showed tumor cells had invaded the pituitary stalk (15/15, 100%). Total resections of CPs with the pituitary stalk were recommended if the pituitary stalk was intraoperatively invaded. In cases where the pituitary stalk was not involved, microsurgical excisions preserving the pituitary stalk were preferred, as there was no significant increase in the recurrence rate and the patients experienced less endocrine dysfunction.

## Introduction

Craniopharyngiomas (CPs) are non-glial tumors and account for 2–5% of all intracranial tumors (1–3), constituting 6–9% of all childhood intracranial tumors (4–6). The age of onset presents bimodal distribution, mainly in 5–15 and 45–55-year-old individuals (5,7) and does not have a significant gender difference. Based on histopathology, CPs are benign tumors. However, the manifestation of CPs is malignant in behavior due to the location of the tumors. CPs are usually located adjacent to the hypothalamus, optical nerve, pituitary stalk, pituitary gland and Circle of Willis. Involvement of these structures within the tumor often leads to visual disorder, endocrine dysfunction and other manifestations (2,8,9).

The gold standard in the treatment of CPs is gross-total resection (GTR) (10–23). Adjuvant radiotherapy is administered in selected cases of residual or recurrent tumors (10,24–30) but chemotherapy is rarely used (31–33). Postoperative curative results and long-term survival rates are improved when CPs are treated with GTR. The inability to perform GTR during the first microsurgery may lead to tumor regrowth and other clinical issues.

CPs mainly originate from the pituitary stalk, which plays a crucial role in endocrine function and water-electrolyte equilibrium. Pituitary stalk preservation may result in incomplete resection as it is often infiltrated or invaded with tumor tissues. Pituitary stalk resection may induce endocrine dysfunction, disruption of the water-electrolyte balance, diabetes insipidus (DI) and other clinical manifestations. The potential benefits of GTR, regarding pituitary stalk preservation or resection, should outweigh the possible recurrence and postoperative endocrine dysfunction for patients. Thus, the problem of pituitary stalk preservation or resection during CP surgery is a significant issue for neurosurgeons.

There have been a number of preliminary studies with regard to pituitary stalk preservation or resection. A previous study of adult individuals indicated that pituitary stalks should be preserved as much as possible (34), as the recurrence rate is not affected but the patients retain complete anterior pituitary function. By contrast, a previous study of children (35) indicated that total resection of CPs together with the pituitary stalk should be performed as the pituitary stalk has no significant role in endocrine functional recovery. These studies indicate that guidelines for pituitary stalk preservation should be established in a greater number of cases, including adults and children.

The aim of the present study was to determine new guidelines regarding pituitary stalk preservation or resection during microsurgery of CPs. A total of 15 pituitary stalk samples from 2010 onwards were analyzed with an ultra-electron microscope to reveal the degree of infiltration or invasion of tumor cells and the guidelines on pituitary stalk preservation or resection were modified accordingly. In total, 203 cases of CPs in adults and children were simultaneously analyzed on a retrospective basis to investigate the difference in the recurrence rate, endocrine function and incidence of DI between patients in Group A (preservation of the pituitary stalk) and Group B (resection of the pituitary stalk).

## Materials and methods

### Patients

Between 1992 and 2012, 203 patients with histopathologically confirmed cases of CP underwent surgery. The gender and age of each patient and the origin, size and location of the tumor, as well as the clinical features, were recorded. All preoperative or postoperative procedures were conducted in accordance with the Declaration of Helsinki. Ethical approval was provided by The Xiangya Hospital, Central South University (Changsha, China).

### Neuro-imaging

All the patients had undergone preoperative computed tomography (CT) and magnetic resonance imaging (MRI) scans of the head. Certain patients had also undergone digital subtraction arteriography. The size and location of the tumor was recorded. Postoperative CT scans of the head were immediately performed following surgery, while MRI scans with contrast were conducted within 24 h (to exclude any neovascularization that may have mimicked the residual tumor). Repeat MRI scans were performed every 3 months during year 1, every 4 months during year 2, every 6 months during years 3–5 and every year as from year 6. All the data were collected as soon as possible.

### Recommendations for pituitary stalk preservation or resection

Certain principles were observed with regard to the management of the pituitary stalk during the microsurgery of the CPs. Pituitary stalks were preserved completely if the CP had an intrasellar origin. Of the 165 cases of CPs originating from the pituitary stalk, the following three criteria were considered for complete resection. Firstly, the origin of the CP was the pituitary stalk and all parts were incorporated within the tumor. Secondly, the pituitary stalk macroscopically showed a significant thickened sausage-like appearance and the volume had significantly increased. Finally, the color of the pituitary stalk was grayish-white, indicating deprivation of the blood supply.

### Postoperative analysis of the pituitary stalk under an ultra-electron microscope

From 2010 onwards, 15 specimens of resected pituitary stalk together with the tumor were examined using an ultra-electron microscope (H-7500 transmission electron microscope; Hitachi Company, Shiga, Japan) to determine whether the tumor was invasive or if infiltration was present.

### Patient grouping and postoperative outcomes

Detailed case histories and intraoperative observations of the patients were recorded carefully. Long-term follow-ups, regarding long-term survival, tumor recurrence, endocrine status, visual acuity (VA) and visual field (VF), were carried out between 1992 and 2012. The shortest follow-up time was 3 months while the longest was 20 years, with an average time of 53.4±47.2 months.

According to the conditions of pituitary stalk preservation, patients were divided into Group A (preservation of the pituitary stalk) and Group B (resection of the pituitary stalk). Differences in the recurrence rate and endocrine functions between the two groups were investigated.

### Statistical analysis

The starting point of overall survival (OS) was defined as the day the patients had surgery and the terminal point was defined as the day the patients succumbed to their illness. The starting point of progression-free survival (PFS) was defined as the day that patients had surgery and the terminal point was defined as the day that the tumor recurrence or regrowth was detected. Kaplan-Meier and logrank tests were used to analyze the OS and PFS. χ^2^ and Fisher’s exact tests were used to compare the data between the two groups. P<0.05 was considered to indicate a statistically significant difference. All statistical data were calculated with Office 2003 (Microsoft Corporation, Redmond, WA, USA) and SSPS version 15.0 (SSPS, Inc., Chicago, IL, USA).

## Results

### Patients

**Figure 1:**
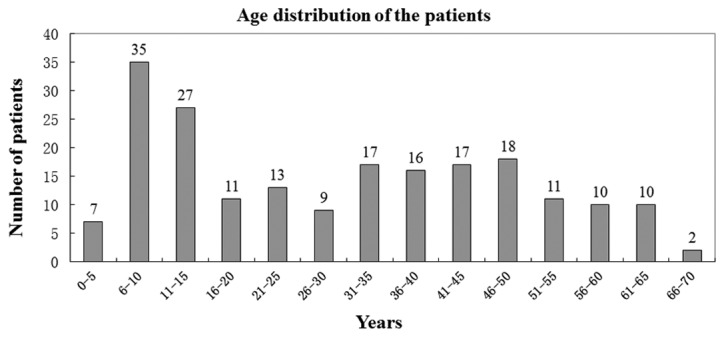
Age distribution of the patients.

Between 1992 and 2012, 203 patients underwent surgery by the same surgeon and their histopathological examinations confirmed the diagnosis of CPs. Of these 203 patients, 128 cases (63.1%) were adults (male, 75; female, 53) while 75 cases (36.9%) were children (male, 45; female, 30). The youngest patient was 3.5 years old and the oldest was 70 years old, and the average age of the patients was 30.1±18.45 years. The age distribution of the patients is shown in Fig. 1.

Among the 203 patients, there were 193 cases of primary tumors and 10 cases of recurrent tumors. Four patients were required to undergo secondary surgery in view of tumor progression. A total of 207 surgeries were conducted.

In the study, tumor sizes ranged between 10 and 90 mm and were further subdivided into 4 subgroups: 11 cases, <2.0 cm; 118 cases, 2.0–4.0 cm; 64 cases, 4.0–6.0 cm; and 14 cases, >6.0 cm. The average tumor size was 38.6±14.0 mm.

Among the 203 patients, 165 cases originated from the pituitary stalk, 36 cases sourced from the sellar area and 2 cases had an ectopic origin (cerebellopontine angle and optic chiasm).

The 203 patients were assigned by tumor location according to Yaşargil’s classification (12), as depicted in Table I. The clinical features of the 203 patients are described in Table II.

### Surgical approaches and results

The subfrontal-lamina terminalis approach was the most frequently used. According to the tumor locations, the surgical approaches varied. Trans-sphenoidal surgery was only used once in the study for the case of a sellar CP. The surgical approaches that were used are listed in Table III.

**Figure 2:**
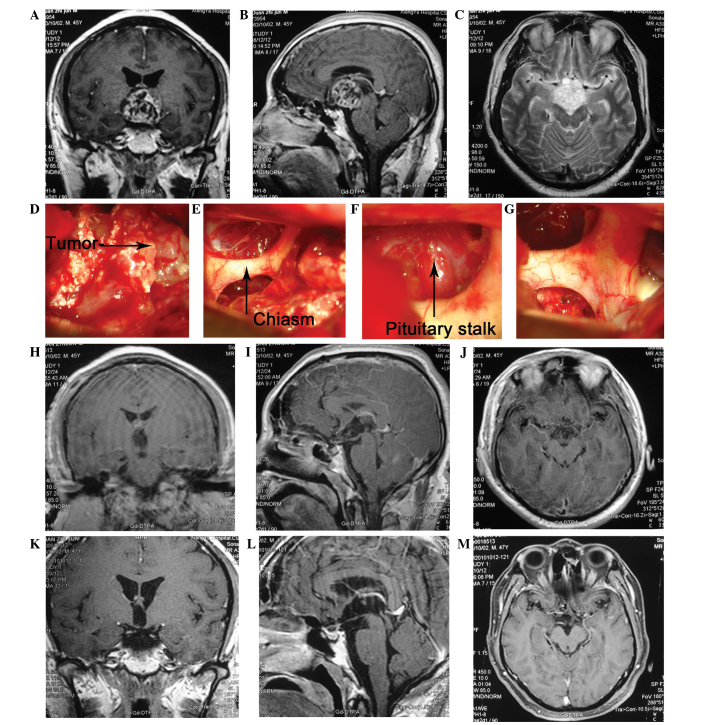
Case of a 45-year-old male who presented with general fatigue for two months, polyuria with blurred vision and memory loss for >1 month. The patient was diagnosed with a CP. During surgery, the tumor was found to originate from the pituitary stalk. In order to completely remove the tumor, the pituitary stalk was also removed. Three years following surgery, the patient resumed work and there was no evidence of recurrence in MRI scans. (A) The coronary view of CE MRI showing that the tumor was located in the sellar and parasellar area. The lesion is mainly solid with contrast enhanced walls. The borders of the tumor are distinct with absence of peritumoral edema. Mild compression of the right side of the third ventricle can also be noted. It also demonstrates the pituitary stalk, the optic nerve and optic chiasm (see arrow). (B) The sagittal view of CE MRI and (C) axial view, shows the relationship between the tumor and the clinoid part of the ICA, optic nerve as well as optic chiasm. (D, E, F and G) The intra-operative findings of the lesion: the tumor was located retrochiasmatically and below the clinoid part of ICA. The pituitary stalk was invaded by the tumor (see arrow). (H, I and J) Post operative CE MRI depicts complete excision of tumor together with absence of hemorrhage. (K, L and M) There was no evidence for tumor recurrence in the 1 year follow up. CP, craniopharyngioma; MRI, magnetic resonance imaging.

During the >20 years of the present study, 203 patients underwent microsurgery and 175/203 (86.2%) had GTRs. Between 1992 and 2002 (first stage) GTR accounted for only 19/36 cases (52.8%); however, there was an abrupt increase in the GTR rate during the second stage between 2003 and 2012 with 156/167 GTR cases (93.4%). A typical case is shown in Fig. 2. There was a significant difference in the GTR rate between the two stages (χ^2^, 37.78; P<0.05), indicating that in the hands of a skilled surgeon, patients suffering from CPs are likely to have an improved surgical outcome since the senior author has a higher GTR rate.

The perioperative period was defined as the 3 months following surgery. Perioperative mortality was 4/203 (1.97%). One patient succumbed to hypothalamus dysfunction, resulting in hypernatremia, hyperglycemia and coma 35 days following surgery. A second patient succumbed to cardiopulmonary arrest and bilateral dilated pupils 15 days following surgery, despite having a ‘clean’ head CT scan (there was not any focal intracranial hemorrhage and the tumor bed was clear). The third patient succumbed to uncontrollable hyperpyrexia that developed suddenly 5 days following surgery. The cause of mortality of the fourth patient was unknown. The mortalities occurred during the early perioperative period.

### Surgical complications

Postoperative complications were present in the series, particularly electrolyte disturbances and DI. Detailed postoperative complications are shown in Table IV.

### Long-term survival

**Figure 3:**
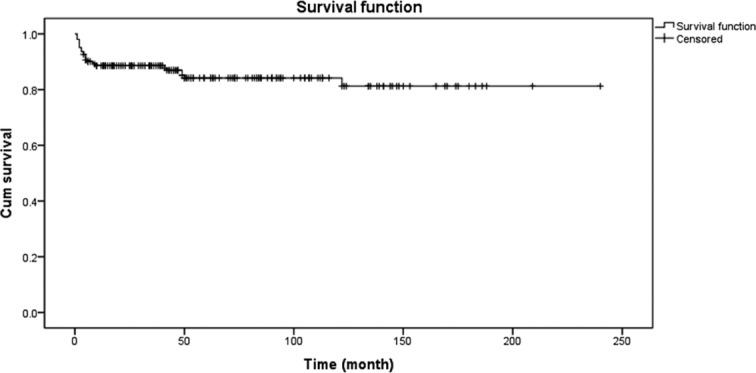
Long-term survival rate curve of the patients. OS was 81.3% and the 5-year survival rate was 84.2%. OS, overall survival.

**Figure 4:**
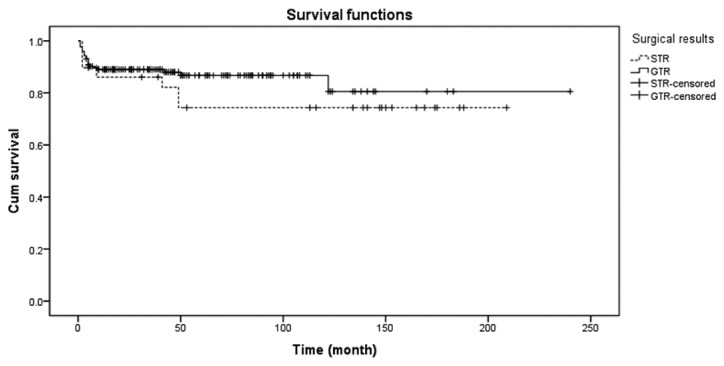
Long-term survival rate curves of GTR and STR patients. (Logrank test, 4.194, P<0.05). GTR, gross-total resection; STR, subtotal resection.

The 5-year survival rate was 84.2% and OS was 81.3%. Details are shown in Fig. 3. There was a significant difference in the long-term survival rate between the GTR and subtotal resection (STR) groups, as shown in Fig. 4.

### Examinations of resected pituitary stalk specimens with an electron microscope

**Figure 5:**
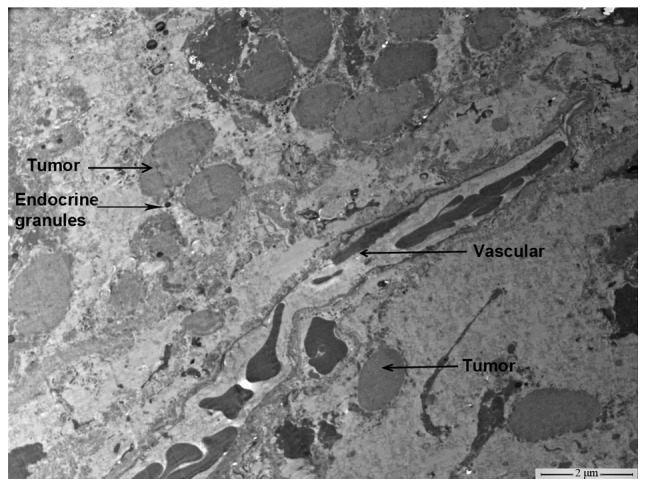
Ultra-electron microscope image of a pituitary stalk demonstrating the invasion of a tumor.

In total, 15 specimens of resected pituitary stalk, which satisfied the aforementioned criteria, were examined postoperatively under an electron microscope to determine whether the samples were invaded by tumor cells. The results showed that tumor cells were present in all the 15 resected pituitary stalks (100%). Fig. 5 shows an electron microscope image. The pituitary stalk is the unique intracranial structure that contains longitudinal blood vessels and tissues. The karyoplasmic ratio in these cells was higher indicating the presence of tumor cells. The regular morphology indicated the benign nature of CPs, while the secretory granules located in the surroundings indicated its endocrine nature. The electron microscope images clearly indicated that all the pituitary stalk specimens were invaded with tumor cells. Therefore, it is necessary to remove the pituitary stalk completely as imminent tumor regrowth is likely otherwise.

### Tumor recurrence

Tumor recurrence was analyzed in the 157 patients who had undergone GTR and were followed-up on a long-term basis. The recurrence rate of patients in Group A (4/34, 11.8%) was not significantly different from that of Group B (10/123, 8.1%; P>0.05). Of these 157 CP cases, 128 cases originated from the pituitary stalk. In addition, no significant difference was identified in the recurrence rate between Group A (1/19, 5.3%) and Group B (6/109, 5.5%; P>0.05), even when the origin of the CPs was considered.

### Progression rates of tumors following GTR and STR

**Figure 6:**
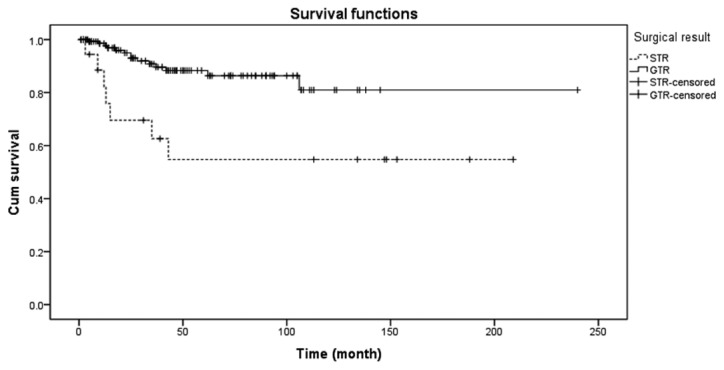
Progression-free survival rate curve of patients who underwent GTR or STR. (Logrank test, 10.198; P<0.05). GTR, gross-total resection; STR, subtotal resection.

**Figure 7:**
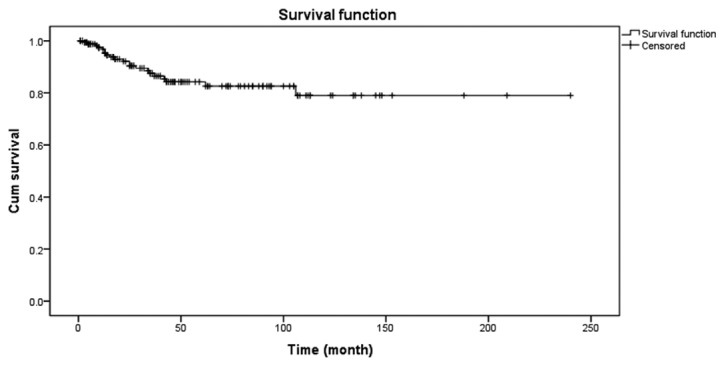
Progression-free survival rate curve of CP patients. PFS at 5 years was 84.2%. PFS, progression-free survival; CP, craniopharyngioma.

In the series, 157 patients received GTR and there were 14 cases of recurrence with a rate of 14/157 (8.9%). With regard to the 21 patients who underwent STR, 7 cases of regrowth were observed with a rate of 7/21 (33.3%). There was a significant difference between the two groups, as shown in Fig. 6 (P<0.05). The mean progression rate of the 178 patients was 21/178 (11.8%), as shown in Fig. 7.

### Treatment of recurrent and residual tumors

In the cases of recurrence in patients that had undergone GTR, re-GTR, GTR + intracavitary irradiation (RT) with phosphorus (^32^P), STR + intracavitary RT with ^32^P, intracavitary RT with ^32^P, γ knife surgery (GKS) and observation were the possible treatment methods. As for patients treated with STR, the possible treatments were GTR, re-STR, re-STR + GKS, GKS, RT and observation. The preferred treatment methods and the number of patients treated with each are illustrated in Table V.

### Endocrine function

Due to incomplete information and loss of follow-up data, there were 129 cases with a known endocrine status prior to surgery, 127 cases 1 week following surgery, 109 cases 3 months postoperatively, 91 cases 1 year following surgery and 91 cases at the last follow-up, as shown in Table VI.

Endocrine status was defined as normal (entire endocrine axis was normal), satisfactory (1 or 2 endocrine axes were abnormal) or poor (≥3 endocrine axes were abnormal). Table VII summarizes the endocrine statuses of the patients in Group A and B. The endocrine status in patients who had undergone GTR or STR was evaluated to investigate any differences with respect to the extent of surgical decompression. Table VIII summarizes the endocrine status in the GTR and STR groups.

### DI incidence rate

Table IX summarizes the DI incidence rates in Group A and B.

### Outcome of VA and VF

Short- and long-term outcomes of postoperative VA and VF are summarized in Table X. In total, 42 patients (55.3%) experienced an improvement in VA and 17 patients (29.3%) had an improved VF status in the short-term following surgery. However, 5 patients (16.1%) with normal VA and 8 patients (10.5%) with abnormal VA experienced a deterioration. The deterioration of VF perimetry occurred in 19 patients (38.8%) with normal VF and 22 patients (37.9%) with abnormal VF.

In the long-term follow-up, 46 patients (60.5%) with abnormal preoperative VA experienced an improvement. In addition, 19 patients (32.8%) with abnormal preoperative VF showed an improved VF perimetry. However, deterioration in VA was observed in 6 patients (19.4%) with normal VA and 10 patients (13.2%) with abnormal VA, and deterioration of VF occurred in 21 patients (42.9%) with normal VF and 25 patients (43.1%) with abnormal VF.

## Discussion

The origin of CPs is quite obscure despite having a close association with the pituitary gland and stalk. Two hypotheses have been reported: The embryo-genetic and the metaplastic theories (36). The embryo-genetic theory hypothesizes that CPs originate from the distal portion of the adenohypophysis, in particular the craniopharyngeal canal or remnant epithelium on Rathke’s cysts. The second theory stipulates that CPs originate from metaplastic epithelial squamous cells of the primitive oral cavity; namely from pars tuberalis of the adenohypophysis. The distal portion of the adenohypophysis forms part of the pituitary gland, whereas the pars tuberalis is an integral part of the pituitary stalk. Thus, this is the theoretical basis for defining intrasellar or pituitary stalk origin.

The origin of CPs has a close association with the pituitary stalk. Therefore it is particularly important to identify this structure during microsurgery. The pituitary stalk is easily identifiable under normal conditions. However, under a pathological state, it is difficult to identify due to the distortion of its shape, displacement or incorporation within the tumor. Based on a previous study (37), the following methods were designed to identify the pituitary stalk. The first relates to the anatomical position of the pituitary stalk. It is connected upwards by the hypothalamus and courses downwards through the diaphragma sellae foramen to enter the intrasellar region and link with the pituitary gland. During microsurgery, the pituitary stalk is identified as the structure running through the diaphragma sellae foramen connecting the hypothalamus and pituitary gland. Secondly, the pituitary stalk may be identified by its unique microscopic structure. Longitudinal stria medullaris structures are observed on the surface of the pituitary stalk. The pituitary stalk is a unique intracranial structure that contains longitudinal blood vessels and tissues. Any structure found in the sellar area bearing these characteristics should be considered as the pituitary stalk.

Since the pituitary stalk has a close association with CPs, management of the pituitary stalk is a significant issue during microsurgical excision. However, to date there have only been a few studies concerning the identification and preservation of the pituitary stalk. The majority of studies (12,34,38–42) indicate whether craniotomy or trans-sphenoidal surgery is the most optimal surgical route to identify as much of the pituitary stalk as possible (Table XI). In addition, the studies indicate that whenever the pituitary stalk is involved within the tumor, it can be resected to achieve GTR. Moreover, when the pituitary stalk is not invaded or partially infiltrated by tumor cells, it should be preserved to outweigh the risk of endocrine dysfunction and DI. Preservation of the distal end of the pituitary stalk only is likely to alleviate DI and be beneficial for adenohypophysis recovery. Finally, the studies indicate that pituitary stalk preservation guarantees the integrity of the hypothalamus.

Two contradictory views exist regarding the preservation of the pituitary stalk. One study of 17 children (35) demonstrated that the patients who had undergone microsurgery preserving the pituitary stalk had a higher recurrence rate and poor endocrine functions. The study strongly indicates that pituitary stalk resection to the highest degree appears to be a more radical treatment than pituitary stalk preservation. By contrast, another study considered that preserving the pituitary stalk around the tumor was important, as the patients consider this surgery more tolerable (43).

In the present study, guidelines of pituitary stalk preservation or resection were proposed. In cases where the pituitary stalk was removed, according to the guidelines, tumor cells were detected in all the specimens of the resected pituitary stalk with an ultra-electron microscope (15/15, 100%). These results support the proposed guidelines as being effective and reliable.

Postoperative recurrence and regrowth of CPs is quite common and problematic. The recurrence and regrowth rate varies between 7 and 30% and is directly proportional to the degree of resection, histopathological types and other factors. The vast majority of studies propose GTR for the treatment of CPs as the recurrence rate is relatively low. In cases where patients did not undergo GTR, higher regrowth rates and secondary surgery risks were observed. Currently there are various views regarding the recurrence and regrowth of CPs. Certain studies hypothesize that secondary surgery may be performed in order to achieve total radical cure. Others consider secondary surgery coupled with adjuvant radiation therapy to be preferable for total cure. With regard to the first hypothesis, GTR is a difficult procedure since serious adhesions hinder a clear dissection. The GTR may also induce hypothalamic dysfunction. Secondary surgery coupled with adjuvant RT is a procedure that is prone to complications, including radionecrosis, dementia and radio-induced tumors. In the current study, 14 cases of recurrence were observed in the 157 GTR cases (8.9%), whereas 7 cases of regrowth (33.3%) were recorded out of the 21 cases of STR. Therefore, there is a significant difference regarding tumor progression between GTR and STR patients.

CPs mainly originate from the pituitary stalk and the present study was conducted to determine whether pituitary stalk preservation correlates with tumor recurrence. The study by Jung *et al* (35) indicated that there was no significant difference in the recurrence rate between patients with preserved or resected pituitary stalks. By contrast, Yamada *et al* (43) concluded that a higher GTR rate with a lower pituitary stalk preservation rate results in a low recurrence rate. Therefore, the authors hypothesize that the lower recurrence rate may be relative to the lower pituitary stalk preservation rate. The results of the present study showed that of the 157 GTR patients, there was no significant difference in the recurrence rate between patients with preserved pituitary stalks (4/34, 11.8%) and those with resected pituitary stalks (10/123, 8.1%). Among the 157 patients, 128 cases of CPs originated from the pituitary stalk. There was no statistical difference in the recurrence rate between the preservation (1/19, 5.3%) and the resection groups (6/109, 5.5%). Therefore, we hypothesize that pituitary stalk resection or preservation, based on the aforementioned principles, are not likely to increase the recurrence risk in patients.

Almost all CP patients develop postoperative endocrine dysfunction (44). Endocrine function has a close association with the degree of resection, histopathological subtypes and involvement of the hypothalamus within the tumor. Replacement therapy is generally indicated for patients with endocrine dysfunction. The drugs commonly used are hydrocortisone, thyroxine, sex hormones and vasopressin tannate. In the current study, postoperative endocrine dysfunction was more common than preoperative endocrine dysfunction, as shown by a marked decrease of growth hormone (GH). Prior to surgery, there were 26 cases (20%) of GH decrease, while it increased to 99 cases (78%) postoperatively. The sexual gland axis (LH/FSH, as shown in Table VI) showed no significant reduction, but it was kept at a higher ratio. The sexual gland axis is most easily damaged due to its invasion by CPs or during microdissection by the surgeon.

The pituitary stalk plays a vital role in postoperative endocrine function. Thus, patients with preserved pituitary stalks exhibit less endocrine dysfunction postoperatively. The study by Jung *et al* (34) indicated that pituitary stalk preservation benefits adenohypophysis function recovery. In the present study, endocrine functions in patients with preserved pituitary stalks were superior to those of patients with resected pituitary stalks. Thus, pituitary stalk preservation had a positive significance in the recovery of endocrine function.

Pituitary stalk preservation has a close association with DI. Therefore, it is important to preserve the pituitary stalk. Even partial preservation of the pituitary stalk is important for the production of antidiuretic hormone postoperatively. Honegger *et al* (38) reported that the incidence of DI in patients with resected pituitary stalks was higher compared with that in patients with total preservation of the pituitary stalk, which was also observed in the present study. The incidence rate of DI revealed no significant difference according to pituitary stalk treatment during the early postoperative stage. However, the long-term incidence of DI in patients with preserved pituitary stalks was significantly lower than that of patients with resected pituitary stalks. Therefore, the pituitary stalk should be actively identified and preserved during sellar region surgery. Partial pituitary stalk preservation may also reduce the long-term incidence of DI. The early recovery conditions for DI in cases with preserved pituitary stalks were significantly better compared with those of cases with seriously involved or partially preserved pituitary stalks, indicating that the protection of the pituitary stalk is extremely important for promoting the function of the hypothalamus-pituitary-endocrine axis. These results were also concordant with the hypothesis by Honegger *et al* (39) that the postoperative probability of DI in patients with preserved pituitary stalks was lower compared with that of patients with resected pituitary stalks.

Postoperative VA was found to improve in more cases than it deteriorated. However, postoperatively there were more patients with decreased VF than improved VF, indicating that CPs have an inevitable effect on VF. The reduction of VF may be explained by the blood vessels supplying visual organs being invaded by the tumor or being sacrificed during resection (45). Thus, a greater number of patients presented with VF defects.

In conclusion, the total resection of CPs together with the pituitary stalk is recommended if intraoperatively the pituitary stalk is found to be invaded. Postoperatively, with the aid of an ultra-electron microscope, all the specimens were observed to be invaded by tumor cells. In cases where the pituitary stalk is not involved, microsurgical excision preserving the pituitary stalk is preferred as there is no significant increase in the recurrence rate and patients exhibit less endocrine dysfunction.

## Figures and Tables

**Table I:** Tumor location and distribution.

Location	Cases, n (%)
Intrasellar	1 (0.49)
Suprasellar	44 (21.67)
Intra-suprasellar	45 (22.17)
Intra-suprasellar-3rd ventricular	29 (14.00)
Suprasellar-3rd ventricular	82 (40.39)
3rd ventricular	1 (0.49)
Ectopic (cerebellopontine angle)	1 (0.49)

**Table II:** Clinical symptoms.

Manifestation	Cases, n (%)
VA impairment	141 (69.5)
VF defect	107 (51.7)
Headache	103 (50.7)
Polydipsia and polyuria	71 (35.0)
Nausea and vomiting	59 (29.0)
Growth retardation (children)	42 (56.0)^a^
Fatigue	39 (19.2)
Decreased libido (adults)	34 (26.6)^b^
Irregular menstruation (female adults)	27 (50.9)^c^
Epileptic fits	11 (5.3)
Galactorrhea	5 (2.5)

a No. of children, 75; 42/75, 56%.

b No. of adults, 128; 34/128, 26.6%.

c No. of female adults, 53; 27/53, 50.9%.

VA, visual acuity; VF, visual field.

**Table III:** Various surgical approaches employed during microsurgery of CPs.

Surgical approaches	Surgeries, n (%)
Subfrontal-lamina terminalis	103 (49.76)
Subfrontal-preoptic	76 (36.71)
Subfrontal-optic nerve-ICA	16 (7.73)
Pterional-preoptic	4 (1.93)
Pterional-optic nerve-ICA	3 (1.45)
Transcallosal	2 (0.97)
Retrosigmoid approach	2 (0.97)
Trans-sphenoidal	1 (0.48)

CPs, craniopharyngiomas; ICA, internal carotid artery.

**Table IV:** Surgery-associated complications.

Complications	Cases, n (%)
Electrolyte imbalance	142 (70.0)
DI	90 (44.3)
Chest infection	6 (3.0)
Central hyperthermia	4 (2.0)
Deep vein thrombosis	4 (2.0)
Disorders of consciousness	3 (1.5)
Subdural effusion	2 (1.0)
Intracranial infection	2 (1.0)
Hypothalamus dysfunction	2 (1.0)
Intracranial hematoma	2 (1.0)
Epilepsy	2 (1.0)
Aneurysm of the right ICA	1 (0.5)^a^

Certain patients developed several complications simultaneously.

a For this patient, saccular aneurysm was found in the right ICA during secondary surgery performed due to tumor recurrence.

DI, diabetes insipidus; ICA, internal carotid artery.

**Table V:** Treatment of residual tumors and recurrent CPs in 35 patients following resection.

Treatment	Patients, n
Treatment of tumor recurrence after GTR	14
Repeat GTR	2
GTR + intracavitary RT (^32^P)	1
STR + intracavitary RT (^32^P)	1
Intracavitary RT (^32^P)	1
GKS	2
None	7
Treatment of residual tumor after STR	21
GTR	1
STR	1
STR + GKS	2
GKS	8
RT	4
None	5

CP, craniopharyngioma; GTR, gross-total resection; STR, subtotal resection; P, phosphorus; GKS, γ knife surgery; RT, radiation therapy.

**Table VI:** Endocrine status at specified intervals.

Endocrine status	Pre-op, n (%) n=129	1 week, n (%) n=127	3 months, n (%) n=109	1 year, n (%) n=91	Last follow-up, n (%) n=91
LH/FSH deficient	88 (68)	92 (72)	77 (71)	58 (64)	59 (65)
GH deficient	26 (20)	99 (78)	72 (66)	53 (58)	50 (55)
TSH deficient	52 (40)	100 (79)	77 (71)	57 (63)	54 (59)
ACTH deficient	65 (50)	101 (80)	80 (73)	63 (69)	64 (70)
Hyper PRL	26 (20)	51 (40)	45 (41)	29 (32)	28 (31)
DI	45 (35)	89 (70)	66 (61)	39 (43)	37 (41)

DI, diabetes insipidus; GH, growth hormone; TSH, thyroid-stimulating hormone; LH, luteinizing hormone; FSH, follicle-stimulating hormone; ACTH, adrenocorticotropic hormone; PRL, prolactinemia.

**Table VII:** Association between endocrine status and groups A and B.

	Neuro-endocrine function			
	
Patients	Normal, n (%)	Satisfactory, n (%)	Poor, n (%)	Total, n
Group A	5 (21.7)	18 (78.3)	0	23
Group B	1 (1.5)	60 (88.2)	7 (10.3)	68
Total	6 (6.6)	78 (85.7)	7 (7.7)	91

Fisher’s Exact test (χ^2^, 10.895; P<0.05).

**Table VIII:** Association between the degree of resection and endocrine function.

	Neuro-endocrine function			
	
Extent of resection	Normal, n (%)	Satisfactory, n (%)	Poor, n (%)	Total, n
GTR	6 (7.1)	73 (85.8)	6 (7.1)	85
STR	0	5 (83.3)	1 (16.7)	6
Total	6 (6.6)	78 (85.7)	7 (7.7)	91

Fisher’s Exact test (χ^2^, 1.328; P>0.05). GTR, gross-total resection; STR, subtotal resection.

**Table IX:** Association between DI and Group A/B.

	DI	
	
Patients	Yes, n (%)	No, n (%)
Group A	5 (16.1)	26 (83.9)
Group B	44 (37.3)	74 (62.7)
Total	49 (32.9)	100 (67.1)

(χ^2^=4.98, P<0.05). DI, diabetes insipidus.

**Table X:** VA and VF recovery for the patients who received follow-ups.

		Short-term outcome, n (%)			Long-term outcome, n (%)		
			
Variable	Patients, n	Improved	Unchanged	Worsened	Improved	Unchanged	Worsened
VA							
Pre-op normal	31	0	26 (83.9)	5 (16.1)	0	25 (80.6)	6 (19.4)
Pre-op abnormal	76	42 (55.3)	25 (32.9)	8 (10.5)	46 (60.5)	20 (26.3)	10 (13.2)
Overall	107	42 (39.3)	51 (47.7)	13 (12.1)	46 (43.0)	45 (42.1)	16 (15.0)
VF							
Pre-op normal	49	0	30 (61.2)	19 (38.8)	0	28 (57.1)	21 (42.9)
Pre-op abnormal	58	17 (29.3)	19 (32.8)	22 (37.9)	19 (32.8)	14 (24.1)	25 (43.1)
Overall	107	17 (15.9)	49 (45.8)	41 (38.3)	19 (17.8)	42 (39.3)	46 (43.0)

VA, visual acuity; VF, visual field.

**Table XI:** Key studies and views associated with the identification and preservation of the pituitary stalk.

Author	Date	Total cases, n	Cases involved in calculation, n	Pituitary stalk identification, n (%)	Pituitary stalk preservation, n (%)	Views
Yaşargil	1990	144	115	74 (64.3)	42 (36.5)	Pituitary stalk preserved as much as possible
Honegger	1999	143	127	74 (58.3)	69 (54.3)	Pituitary stalk preserved as much as possible
Van Effenterre	2002	122	122	122 (100.0)	64 (52.5)	Pituitary stalk preserved as much as possible
Nishizawa	2006	22	22	11 (50.0)	2 (9.1)	Tumor resected to the maximum extent
Stamm	2008	7	7	7 (100.0)	4 (57.1)	Pituitary stalk preservation
Shi	2008	309	309	235 (76.1)	186 (60.2)	Pituitary stalk preserved as much as possible
Jung	2009	41	39	NA	24 (61.5)	Pituitary stalk preserved as much as possible
Jung	2010	17	17	17 (100.0)	7 (41.2)	Tumor resected to the maximum extent
Yamada	2010	90	52	52 (100.0)	16 (30.8)	Tumor resected to the maximum extent
Current study	2012	203	203	152 (74.9)	34 (16.7)	Tumor resected to the maximum extent if resection standards are achieved or preservation.
